# The signed Kolmogorov-Smirnov test: why it should not be used

**DOI:** 10.1186/s13742-015-0048-7

**Published:** 2015-02-27

**Authors:** Guillaume J Filion

**Affiliations:** 1 Genome Architecture, Gene Regulation, Stem Cells and Cancer Programme, Centre for Genomic Regulation (CRG), Dr. Aiguader 88, 08003 Barcelona, Spain; 2 Universitat Pompeu Fabra (UPF), Barcelona, Spain

**Keywords:** Kolmogorov-smirnov test, Statistics, P-value, P-hacking

## Abstract

The two-sample Kolmogorov-Smirnov (KS) test is often used to decide whether two random samples have the same statistical distribution. A popular modification of the KS test is to use a signed version of the KS statistic to infer whether the values of one sample are statistically larger than the values of the other. The underlying hypotheses of the KS test are intrinsically incompatible with this approach and the test can produce false positives supported by extremely low p-values. This potentially makes the signed KS test a tool of p-hacking, which should be discouraged by replacing it with standard tests such as the t-test and by providing confidence intervals instead of p-values.

## Background

From its inception, the two-sample Kolmogorov-Smirnov (KS) test was designed as a generic method to test whether two random samples are drawn from the same distribution. The null hypothesis of the KS test is that both distributions are identical, without any further assumption regarding their location and shape, which makes the KS test widely applicable. The statistic of the KS test is a distance between the two empirical distributions, computed as the maximum absolute difference between their cumulative curves. Several studies in the field of genomics (such as [1-5]) have suggested the use of the signed difference between the cumulative curves. According to this view, the sign of the statistic indicates which of the two distributions has the larger values. This procedure does not have a formal name; for clarity, I will refer to it as the “signed KS test” (sKS test).

**Figure 1:**
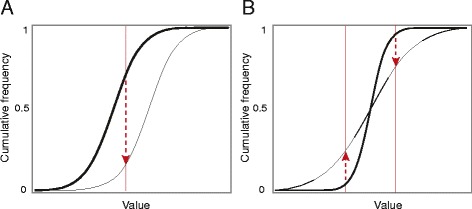
**Comparison of ideal samples by the signed KS test. (A)** The distributions have different locations. The lines represent the empirical cumulative distributions of each sample (the reference sample is plotted as a bold line). The KS statistic is the maximum vertical distance between the curves and is indicated by the vertical red line. As the reference sample is on the left, the arrow points downwards, so the statistic is negative. **(B)** The distributions have different variances. In this example there are two positions where the vertical distance is at a maximum, indicated by the two red lines. As the arrows point in opposite directions, the sign of the KS statistic is not defined.

The argument for using the sKS test is best represented graphically. Figure 1A shows the example of two distributions compared by the sKS test for two random samples of infinitely large size. The red arrow indicates the maximum difference between the cumulative curves. Taking the bold curve as the reference, the arrow points downwards, which means that the sign of the sKS statistic is negative. If the thin curve was to the left of the bold curve, then the arrow would point in the opposite direction and the sKS statistic would be positive. Therefore the sign of the sKS statistic seems to indicate the sample with the statistically highest values.

However, this argument makes an implicit assumption that does not necessary hold. Figure 1A shows two curves with the same shape, which means that they can differ only by their location, i.e. by a shift to the left or to the right. However, the KS test discriminates distributions when they differ by either their location or by their shape.

Figure 1B shows another ideal example of two distributions compared by the sKS test, but this time they differ only in their variance. There are two positions at which the cumulative curves differ the most, which is why two arrows are drawn. More importantly, one arrow points upward, whereas the other points downward, so that the sign of the sKS statistic is undefined. In finite samples, the distributions are never perfectly symmetrical, so one of these arrows would be the longest and each would have a probability of 0.5. Interestingly, the p-value is extremely small if the samples are large, but the sign of the sKS statistic would be random.

This ideal example never happens in practice. The distributions of biological samples typically differ in shape and location, so the situation shown in Figure 1B is unrealistic. In a real example, the difference between the shapes of the distribution will boost the significance of the sKS test, yielding low p-values even when the difference in location is modest or non-existent.

**Figure 2:**
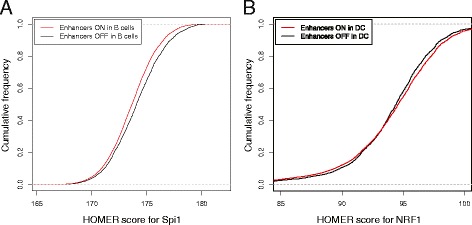
**Use of the signed KS test on real data.** The best HOMER [6] scores of the two motifs are computed for enhancers of the blood lineage and the active enhancers are compared with inactive enhancers in two cell types. **(A)** Comparison of Spi1 motifs in enhancers active in B cells versus inactive enhancers. The curves are shifted relative to each other, which means that the scores are lower overall in the active enhancers. **(B)** Comparison of the NRF1 motifs in the enhancers active in dendritic cells versus inactive enhancers. The curves cross each other, which means that the scores are more variable in active enhancers. However, the medians are very close.

To give a specific example, Figure four (panel C) from Lara-Astiaso et al. [1] is a heat map showing the enrichment of transcription factor motifs computed with the sKS test. The authors compared HOMER [6] scores of 205 motifs in the enhancers that were active in a cell lineage versus the enhancers that were inactive. Following the indications of the authors [1], I have reproduced the data on which the sKS tests were performed and chose two examples out of 3485 (note that I used H3K27ac counts as a proxy for activity as the ATAC counts were not provided). Figure 2A shows the distribution of the scores for the Spi1 motif. The scores of the enhancers active in B cells are lower than those that are inactive, as shown by the horizontal shift between the curves. This example corresponds to Figure 1A, where the sKS test is meaningful. For comparison, Figure 2B shows the distribution of scores for the NRF1 motif. In this example, the cumulative distributions cross each other, as in Figure 1B. This means that, in dendritic cells, the active enhancers have more variable scores for the NRF1 motif than the inactive enhancers or, in other words, the high scores are higher and the low scores are lower. Thus the evidence for the depletion of the NRF1 motif is questionable, although the p-value of the KS test is 4.2 × 10^−10^, which would be considered significant by most researchers. In comparison, the p-value of the t-test performed on the same sample is 0.20, which is not significant according to any standard. In summary, the distributions are different, but the interpretation that their means differ is wrong (note that the increased variability may still be relevant from the biological point of view).

If the distributions have the same shape (as in Figure 2A), then the sKS test is meaningful, but there is still no reason to use it because it is less powerful than the t-test and even the Wilcoxon-Mann–Whitney test. In other words, the t-test and the Wilcoxon-Mann–Whitney test have more chance of detecting a shift when it exists (Garrett Jenkinson provides a power analysis in his review of this article, available in the pre-publication history). This issue is due in part to floor and ceiling effects, meaning that the sKS test statistic will be small if it is in either tail of the distribution.

It is thus surprising that an unconventional approach such as the sKS test would be used in place of an established standard such as the t-test. Among other reasons, it may be part of a flawed practice called “p-hacking”, which is to test the same statistical hypothesis in different ways until a target p-value is obtained. The misconception at the root of p-hacking is that a higher statistical significance entails a larger biological response (Figure 2B is an example of the opposite). Replacing the sKS test by more standard options would be an improvement, but a better method can be used.

When using a statistical test to evaluate the significance of a response, it is important to conclude with a statement regarding the magnitude of the effect. Confidence intervals are a natural check that should help researchers distinguish statistical significance from biological significance. For instance, in the example shown in Figure 2A, the t-test yields a p-value lower than 2.2 × 10^−16^, which suggests that, in B cells, Spi1 HOMER scores are different between active and inactive enhancers. However, giving (0.36, 0.53) as a 95% confidence interval for this difference is more informative because it is a specific statement about the magnitude and it allows the reader to decide whether it is biologically relevant.

## Conclusions

At a time when the field of genomics is progressively becoming standardized, it is important to enforce a certain statistical rigor. The sKS is not consistent and it is less powerful than the t-test and the Wilcoxon-Mann–Whitney test, so there is no reason to use it unless carefully justified. More generally, testing a statistical response should include some information about the magnitude of the effect, for instance in the form of a confidence interval. Such practices would provide valuable information to researchers and discourage p-hacking.

## Abbreviations

KS test: Kolmogorov-Smirnov test
sKS: signed Kolmogorov-Smirnov test
